# Does Left Atrial Appendage Amputation During Routine Cardiac Surgery Reduce Future Atrial Fibrillation and Stroke?

**DOI:** 10.1007/s11886-018-1033-4

**Published:** 2018-08-31

**Authors:** Helena Domínguez, Christoffer Valdorff Madsen, Oliver Nøhr Hjorth Westh, Peter Appel Pallesen, Christian Lildal Carrranza, Akhmadjon Irmukhamedov, Jesper Park-Hansen

**Affiliations:** 1 Department of Cardiology, Bispebjerg-Frederiksberg University Hospital, Nordre Fasanvej 57, vej 4, Building 3, 3rd Floor, DK-2000 Frederiksberg, Denmark; 2 Department of Biomedicine, University of Copenhagen, Blegdamsvej 3B, Panum Building 10.5, DK-2400 Copenhagen, Denmark; 3 Department of Heart, Lung and Vascular Surgery, Odense University Hospital, Sdr. Boulevard 29, DK-5000 Odense, Denmark; 4 Department of Cardio-thoracic Surgery, Blegdamsvej 9, 2100 København, Copenhagen, Rigshospitalet Denmark

**Keywords:** Left atrial appendage, Cardiac surgery, Atrial fibrillation, Thromboembolism, Stroke

## Abstract

**Purpose of Review:**

Stroke is the most feared complication of atrial fibrillation. To prevent stroke, left atrial appendage exclusion has been targeted, as it is the prevalent site for formation of heart thrombi during atrial fibrillation.

We review the historic development of methods for exclusion of the left atrial appendage and the evidence to support its amputation during routine cardiac surgery.

**Recent Findings:**

Evidence is not yet sufficient to routinely recommend left atrial exclusion during heart surgery, despite a high prevalence of postoperative atrial fibrillation. Observational studies indicate that electrical isolation of scarring from clip or suture techniques reduces the arrhythmogenic substrate.

**Summary:**

Randomized studies comparing different methods of closure of the left atrial appendage before amputation do not exist. Such studies are therefore warranted, as well as studies that can elucidate whether amputation is superior to leaving the left atrial appendage stump. Potentially, thrombogenic remaining pouch after closure should be addressed.

## Introduction

Does left atrial appendage (LAA) amputation during routine cardiac surgery reduce the risk of stroke and future atrial fibrillation? This question has been posed for more than 150 years [1, 2], since the LAA is recognized as the prevalent site for formation of thrombi during atrial fibrillation (AF) [3–5], with stroke being its most common and potentially devastating thromboembolic complication.

### Atrial Fibrillation and Its Relation to Open-heart Surgery

Occurrence of AF following cardiac surgery is common, with a prevalence of 10 to 65% [6–8], and is associated with prolonged hospitalization and higher risk of mortality [7–10].

Paroxysmal AF after cardiac surgery is often self-limiting [11], and up to 40% of patients with AF are asymptomatic during the arrhythmia [12]. Hence, many patients with unrecognized recurrent AF, who do not receive anti-coagulation after surgery, remain at risk for stroke [13]. This is a growing problem since more elderly patients undergo cardiac surgery [14]. Although the risk of complications during surgery is improving, patients continue to have a high risk of stroke if they have AF [15, 16]. Therefore, multiple approaches are targeted to relieve this burden. That is, to prevent the occurrence of AF [17, 18] and its recurrence after surgery [19–21], and, to reduce the thrombogenic substrate, the left atrial appendage (LAA) is excluded [22–24]. Oral anti-coagulation (OAC) is an efficient method for thromboembolism risk reduction [25]. However, the adequate length of OAC after post-operative AF is still unknown [18, 26]. Importantly, between 30 and 50% of patients are not eligible for OAC due to high risk of bleeding or other contraindications [25, 27]. Furthermore, it is estimated that 16–50% of patients in OAC therapy are not sufficiently anti-coagulated [28–30]. In these patients a highly reliable LAA occlusion would be an attractive alternative as it potentially reduces stroke risk by 50% [31].

### The Left Atrial Appendage and Thrombus Formation

The LAA is a hook-like diverticulum of the left atrium (LA) consisting of one or more lobes with a trabeculated wall due to parallel-running pectinate muscles [32, 33]. In sinus rhythm, the LAA is highly contractile (contracts from its apex toward the base) and the blood flow within the lumen is sufficient to avoid thrombus formation. Contrarily, during AF, the contractility of the LAA is limited and the blood flow within the lumen is reduced creating a hemodynamic ‘dead-space’ [34, 35]. Furthermore, the highly trabeculated wall of the LAA plays an important role in its high thrombogenicity, and increased thrombus formation occurs with smaller LAA orifice and higher number of lobes [36, 37]. Therefore, the LAA is considered the primary source of cardio-embolic stroke in patients with non-valvular AF [25, 38], although thrombi can develop outside the LAA [3]. Accordingly, transesophageal echocardiography (TEE) is the key examination for diagnosing thrombus formation in the LAA [35, 37, 39], and direct current conversion to sinus rhythm has proved to be safe in the absence of thrombi in the LAA during TEE [33, 34, 40–42].

### Historic Perspective on Amputation of the Left Atrial Appendage During Surgery

The first amputations of the LAA in humans [43] were reported almost simultaneously with the results of the procedure in animal experiments [44–46]. After these successful pioneering attempts, they were subsequently performed in addition to mitral commissurotomy, to alleviate the well-known high thrombogenicity in mitral stenosis [38, 47, 48]. Systematic exclusion of the LAA is currently recommended in addition to surgical ablation procedures [21].

Thoracoscopic amputations of the LAA were initially performed by Johnson et al. as a stand-alone procedure in patients with high risk for thromboembolisms, who do not tolerate OAC [25]. Since then, there have been developed minimally invasive approaches to amputate the LAA [19, 49–53]. Nevertheless, the development of safe and effective clip devices for obliteration of the LAA thoracoscopically [54] and intra-operatively [55••, 56], is emerging as a preferred method compared to LAA amputation [57, 58].

### Less Arrhythmogenic Substrate After LAA Exclusion

Persistent AF can originate from the LAA [59] and it has been demonstrated that targeting ablation of the LAA can reduce AF [21, 60]. Therefore, exclusion of the LAA can possibly provide an anti-arrhythmogenic effect, in addition to protection against thromboembolisms. Accordingly, a reduction of atrial dispersion has been demonstrated with LAA ligation in patients with AF [61]. Additionally, clip occlusion [62] and epicardial ligation [63] have shown to provide electric appendage isolation. In a recent study, patients randomized to closure or not closure of the LAA during cardiac surgery, closure by epicardial suture seemed to reduce AF during follow-up (Park-Hansen in press). It is conceivable that amputation of the LAA can have a similar anti-arrhythmogenic effect, but such an effect remains to be studied.

### Hemodynamic Consequences of LAA Amputation

Natriuretic hormones can be secreted from all myocytes but, in normal healthy conditions they are primarily produced in the LAA [64–67]. Levels of natriuretic hormones are elevated in permanent AF [68] and, specially, levels of B-type natriuretic peptide reveal paroxysms after ablation [69–73].

The first successful LAA amputations on healthy dogs in the 1940s [1] were followed by concerns on possible impairment of mechanisms to compensate fluid overload, due to the loss of atrium natriuretic peptides and a reduced stroke volume of the left atrium [44–46]. Despite these concerns, experimental observations in animals do not seem to have clinical importance in humans [74].

Recently, it has been raised concern by the observation that, along with a decrease on the left atrium volume that follows successful AF ablation, there is an increase in the LAA volume, assessed with magnetic resonance scanning [75]. This is a matter of concern, since larger LAA volume increases the risk of stroke in patients with comparable thromboembolic risk profile [76, 77].

In a recent study, no hemodynamic changes have been observed immediately after percutaneous LAA closure [78]. Nevertheless, it seems to be important whether the LAA is closed from the endocardial or from the epicardial site, since comparing the two approaches, only the epicardial closure proved beneficial hemodynamic changes [79].

According to these observations, an epicardial closure, with or without subsequent amputation, should be the preferred approach.

### Thrombogenicity After Amputation of the LAA

The thrombogenicity of the LAA during AF is, in part, attributable to loss of contractility of the LAA. In a retrospective study of patients who underwent occlusion of the LAA, those with preserved LAA had a better contractility estimated with echocardiography, compared with a group where the LAA was amputated; however, this difference did not preclude differences on stroke nor occurrence of AF [80].

**Fig. 1:**
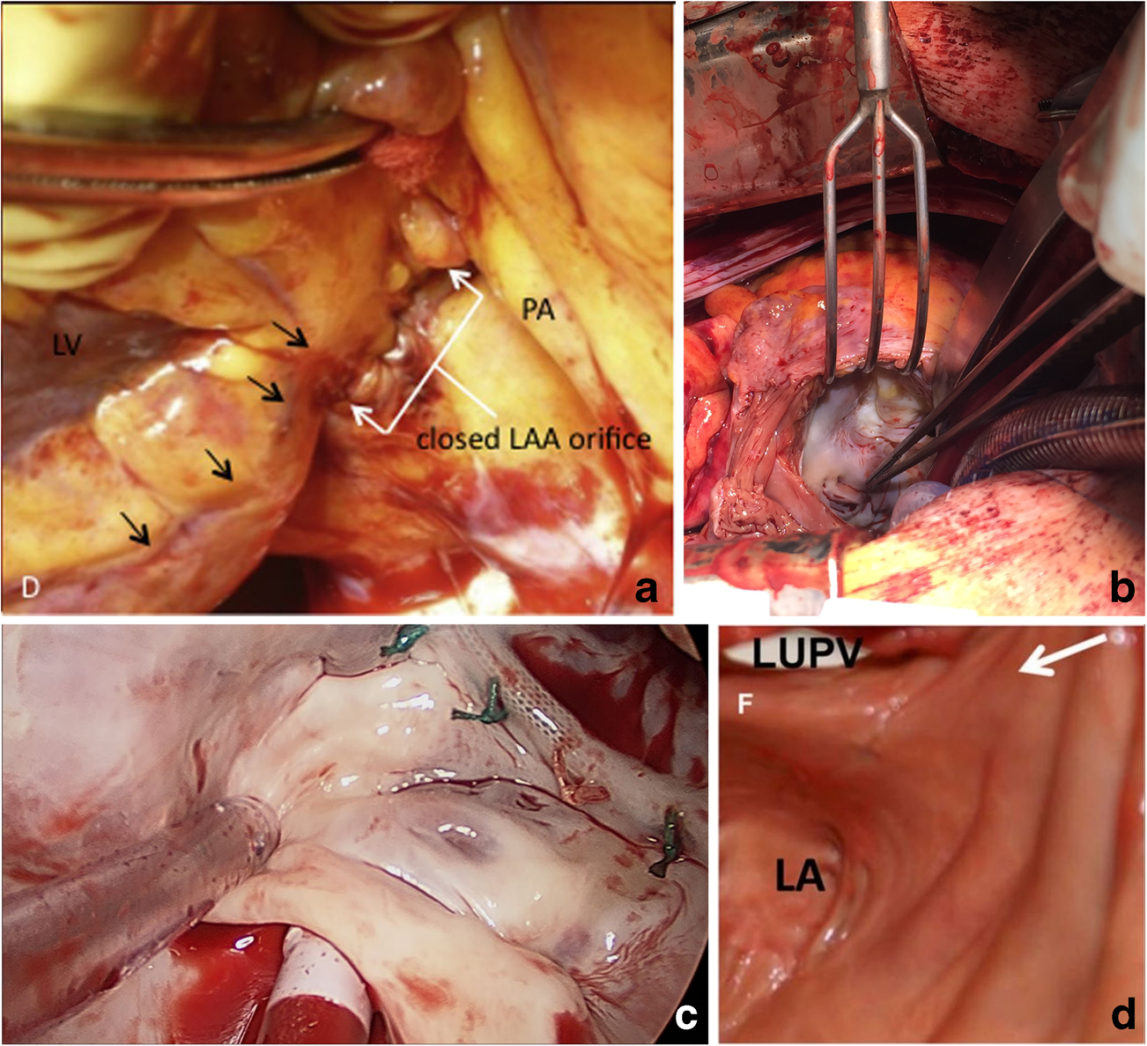
View of the endocardial surface of the left atrium, after LAA exclusion with: suture marked with white arrows, the black arrows show the circumflex coronary artery **a**, epicardial stapling (**b**), clips (**c**), or epicardial snoring, with a black arrow showing the endocardial surface (**d**). *LAA* left atrium appendage. (Figure 1a is reproduced from Aoyagi S. et al. *Heart, Lung and Circulation*, 2017.26:413–15, with permission from Elsevier; [87].) (Figure 1c is kindly provided by AtriCure Inc.) (Figure 1d is reproduced from Bartus K et al. *Circ Arrhythm Electrophysiol.* 2014;7:764–767, with permission; [88])

Regardless of the LAA exclusion method, the potential thrombogenicity of the remaining appendage pouch is a matter of major concern [31, 81••, 82]. In a previous non-randomized study that compared efficacy of several methods of LAA closure, TEE reveled a remnant LAA in 26% of patients. Importantly, 12% of these patients had suffered strokes in the lapse from the operation to the time when TEE was performed, despite none having clots in the remnant LAA [83]. In early studies on excision of the LAA after stapling, additional sutures were necessary to repair tears [84, 85], and similar complications with tears needing repair have also been reported more recently in the LAAOS-II trial [86]. Furthermore, intuitively, an irregular endocardial surface after suture (Fig. 1a) may be more thrombogenic than a smoother surface observable after epicardial stapling (Fig. 1b) and after epicardial clips (Fig. 1c) or snaring (Fig. 1d). Assuming the size of the remaining pouch is important for its thrombogenicity, perioperative TEE could serve for assessing its size, and perioperatively, limit the size of the pouch for example to less than 1 cm, by performing an additional suture or stapler line, or placing an additional clip when needed.

## Conclusion

Several studies support a beneficial effect of LAA closure during surgery, but evidence is not yet sufficient to support closure of the LAA systematically in addition to heart surgery, to protect against thromboembolisms related to AF. Hence, there is a need for randomized studies to provide the evidence of stroke protection and, furthermore, comparing different methods for closure of the LAA in terms of arrhythmogenic impact. Such studies should address how to manage the potential thromboembolic problem of a remaining pouch after LAA closure, to elucidate what is the optimal management of the LAA during surgery.

## Abbreviations

AF: Atrial fibrillation
LA: Left atrium
LAA: Left atrial appendage
OAC: Oral anti-coagulants
TEE: Trans-esophageal echocardiography
